# Simultaneous Electrochemical Detection of Cu^2+^ and Zn^2+^ in Pig Farm Wastewater

**DOI:** 10.3390/s24082475

**Published:** 2024-04-12

**Authors:** Jia-Xin Du, Yang-Hao Ma, Said Nawab, Yang-Chun Yong

**Affiliations:** Biofuels Institute and Institute for Energy Research, School of Environment and Safety Engineering, Jiangsu University, 301 Xuefu Road, Zhenjiang 212013, China; 14729257987@163.com (J.-X.D.); mayanghao2024@126.com (Y.-H.M.); said@ujs.edu.cn (S.N.)

**Keywords:** pig farm wastewater, heavy metals, electrochemical detection

## Abstract

In recent years, the rapid development of pig farming has led to a large quantity of heavy metal-polluted wastewater. Thus, it was desirable to develop a simple heavy metal detection method for fast monitoring of the wastewater from the pig farms. Therefore, there was an urgent need to develop a simple method for rapidly detecting heavy metal ions in pig farm wastewater. Herein, a simple electrochemical method for simultaneous detection of Cu^2+^ and Zn^2+^ was developed and applied to pig farm wastewater. With a glassy carbon electrode and anodic stripping voltammetry, simultaneous detection of Cu^2+^ and Zn^2+^ in water was achieved without the need for complicated electrode modification. Furthermore, it was found that the addition of Cd^2+^ can enhance the response current of the electrode to Zn^2+^, which increased the signal by eight times. After systematic optimization, the limit of detection (LOD) of 9.3 μg/L for Cu^2+^ and 45.3 μg/L for Zn^2+^ was obtained. Finally, it was successfully applied for the quantification of Cu^2+^ and Zn^2+^ with high accuracy in pig farm wastewater. This work provided a new and simple solution for fast monitoring of the wastewater from pig farms and demonstrated the potential of electrochemical measurement for application in modern animal husbandry.

## 1. Introduction

Due to rapid population growth and improving living standards, there has been a remarkable increase in the demand for livestock products in Asia. This has led to subsistence agriculture moving toward an intensive commercial production system [1,2,3]. For example, between 1996 and 2015, beef and milk production increased by 11.9 and 13.0 times, respectively [4]. Especially in China, the total value of the livestock industry increased from 35.42 billion yuan in 1980 to 297.84 billion yuan in 2015 [5]. Consequently, China has emerged as one of the world’s foremost leaders in large-scale livestock and poultry farming. Rapid livestock and poultry farming development has provided plenty of agricultural products. However, it also produced a considerable amount of solid or liquid pig farm waste, leading to serious environmental issues and public health problems [6,7,8].

Heavy metal pollution was one of the main environmental problems in agricultural pollution [9,10]. Modern animal husbandry uses heavy metal additives extensively in animal feeds. Then heavy metals were used as fertilizers through pig farm or farm wastewater, which was discharged indiscriminately, causing severe environmental pollution to soil or water bodies. These phenomena were particularly evident in areas of intensive agriculture. Therefore, intensive livestock farming has been considered an important source of environmental heavy metal pollution [11,12,13].

Heavy metals could damage the health of plants, animals, humans, and the environment. It was challenging to degrade and eliminate because of the persistence and stability of heavy metals. Heavy metals are bioaccumulative and may slowly enter the food chain, with animals ingesting heavy metals through polluted food and accumulating these metals in meat. Excessive accumulation of heavy metals could lead to severe environmental pollution and health risks for organisms [14,15,16]. For example, to reduce bacterial resistance to antibiotics, the European Union banned antibiotics in feed in 2003 [17]. To reduce the cost of farming, pig factories widely added high doses of heavy metals such as zinc oxide, copper salts, and cadmium to feed to promote growth and prevent infections, similarly to antibiotics [18]. However, most of the heavy metals in the feed were transferred to pig farm wastewater or fecal matter. The main components of wastewater were animal discharges and wash treatment water. In the process of livestock and poultry farming, a large number of pollutants will be produced, and the direct discharge of untreated swine manure into surrounding farmland can lead to the enrichment of heavy metals in soil, which, in turn, may lead to the enrichment of heavy metals in crops, posing a potential risk to human health. Cu^2+^, Zn^2+^, and Cd^2+^ contamination of agricultural soils by swine manure has been widely reported [19,20]. The spread of agricultural pollution from livestock and poultry farming has become a serious problem [21,22].

Atomic absorption spectrometry (AAS) [23], inductively coupled plasma atomic emission spectrometry (ICP-MS) [24], microwave-induced plasma optical emission spectrometry (MIP-OES) [25], and inductively coupled plasma optical emission spectrometry (ICP-OES) [26] were the traditional spectroscopic methods used for the analysis of elements. These methods had been applied to detect heavy metal ions with good precision, high sensitivity, and a low detection limit. Unfortunately, the high instrument investment and complicated operation procedures limit their in situ applications in animal husbandry. In contrast, electrochemical detection methods, known for their affordability, high portability, and ease of use, had attracted great interest. Electrochemical analysis was also one of the most promising detection techniques because of its low limit of detection (LOD) and high sensitivity, and it was widely used for the detection of heavy metal ions and various organic compounds [27,28]. Stripping voltammetry, as an electrochemical detection method, was widely utilized for the detection of heavy metals in soil and water samples [29,30,31]. The stripping voltammetry technique consisted of two major steps: accumulation (pre-concentration) and stripping [32]. During the accumulation step, the analyte was deposited at the working electrode surface. The accumulation of analytes through either an oxidation potential, reduction potential, or adsorption to the electrode surface was followed by the measurement of current during an opposite potential scan that reversed the redox process used or deposition. Anodic stripping voltammetry could be used for trace metal analysis to measure multiple metal ions simultaneously in a variety of matrices. In this case, the metal ion (M^+^) was reduced to M by applying a significant negative potential, and then M was oxidized to M^+^, while a peak current proportional to the concentration of M^+^ in solution was recorded. The chemical equation for the use of anodic stripping voltammetry involved in this study could be found in Supporting Information. Moreover, the potential for application of anodic stripping voltammetry in pig farming wastewater is still unknown, which deserves to be investigated.

In this study, an unmodified glassy carbon electrode (GCE) was directly used for simultaneous detection of Cu^2+^ and Zn^2+^ in water by a simple electrochemical approach. The detection of Cu^2+^ and Zn^2+^ in pig farm wastewater was achieved by optimizing the digestion method and detection conditions. In addition, it was found that the electrochemical response to Zn^2+^ was significantly improved by adding Cd^2+^, which greatly enhanced the electrochemical sensitivity for the detection of Zn^2+^. This work not only provides a facile approach for Cu^2+^ and Zn^2+^ monitoring in pig farms but also expands the possibility of future electrochemical detection in animal husbandry for practical applications.

## 2. Materials and Methods

### 2.1. Chemicals and Reagents

Sodium acetate anhydrous, acetic acid, concentrated nitric acid, ammonia, anhydrous ethanol, copper sulfate pentahydrate, zinc sulfate heptahydrate, cadmium chloride dihydrate, lead chloride anhydrous, potassium ferricyanide, potassium chloride, sodium hydroxide, and hydrochloric acid were purchased from Sinopharm Chemical Reagent Co., Ltd. (Beijing, China). All the materials were used without further purification.

Acetate–sodium acetate buffer was prepared with 25 g/L sodium acetate and 10 mL/L acetic acid. The pH was adjusted to 4.8~5.0. For Fenton’s reagent, 1.52 mL of 8% ferrous sulfate was mixed with 0.2 mL of 30% hydrogen peroxide in 100 mL of distilled water, and the pH was adjusted to 2–4 with hydrochloric acid. 

Pig farm wastewater samples were collected from a large-scale pig farm located in Huaian, Jiangsu Province, China, in June 2023. Wastewater is produced during the pig breeding process. The raw wastewater samples were collected before they were discharged into the wastewater treatment plant.

### 2.2. Electrochemical Method

The anodic stripping voltammetry used in this experiment includes square wave stripping voltammetry (SWSV) [33], differential pulse stripping voltammetry (DPSV) [34], and linear scan voltammetry (LSV) [35]. The direction of scanning was from negative potential to positive potential. The working electrode used was a GCE, the reference electrode was a saturated calomel electrode, and the counter electrode was a platinum wire electrode. The CHI660E electrochemical workstation (Shanghai Chenhua, Shanghai, China) was used for all electrochemical measurements. The electrolyte was an acetic acid–sodium acetate buffer at pH 4.8. 

### 2.3. Pre-Treatment Method and Characterization of GCE

The GCE was polished with 1 mm, 0.3 mm, and 0.05 mm aluminum oxide powders on chamois, adding a few drops of deionized water [33]. After polishing, it was rinsed with deionized water to remove residue and ultrasonically cleaned in 50% nitric acid, 50% ammonia, and 50% ethanol, each for 2~3 min. The electrode was then activated in 1 mol/L sulfuric acid, performing CV scanning from −1.0 to 1.0 V until stable curves were observed.

For real sample application, after each sample detection, the GCE was simply polished with different-size aluminum oxide powders and washed three times with deionized water. Then, CV was applied to check the cleanliness of the electrode. 

For GCE observation with scanning electron microscopy (SEM, JSM-7800F, Tokyo, Japan), heavy metal ions were enriched on the electrode, then vacuum-dried at room temperature for 3 min. The electrode head was removed and fixed on a sample stage to observe the morphology of the electrode.

### 2.4. Heavy Metal Digestion 

Several different digestion solutions were used in this study: nitric acid [36], sulfuric acid [37], and hydrogen peroxide [38]. Fenton’s reagent was also used in combination. 1 mL of the digestion solution was added to 10 mL sample solution, and heated for digestion until a clear solution obtained. After cooling, the sample was filtered through a 0.45 mm filter paper and stored for further detection.

### 2.5. Inductively Coupled Plasma Mass Spectrometry

Inductively coupled plasma mass spectrometry (ICP-MS) was used for quantitative detection of different metal ions, as described elsewhere [39]. In brief, 100 mL of the sample was placed into a 250 mL conical flask with 5 mL of HNO_3_ and heated at 95 ± 5 °C for 30 min. The mixture was then cooled and filtered through a 0.45 mm membrane, and the supernatant was used for analysis. The ICPE-9000 (Shimadzu, Tokyo, Japan) was used for metal ion detection, and the internal standard elemental solution was applied.

## 3. Results and Discussion

### 3.1. Development of Electrochemical Method for Simultaneous Detection of Cu^2+^ and Zn^2+^

**Figure 1:**
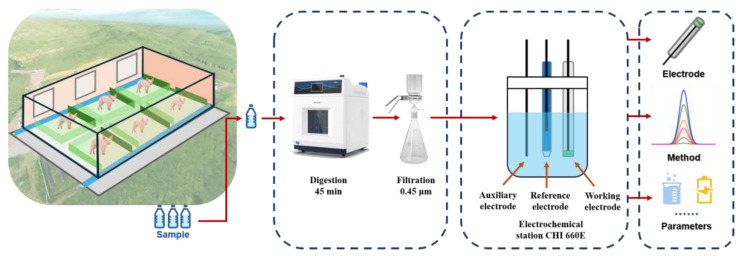
Schematic for electrochemical detection of heavy metals in pig farm wastewater.

Owing to the high turbidity and high organic content of pig farm wastewater, samples needed to be digested before electrochemical detection. After the digestion, different anodic stripping voltammetry methods were used to detect the heavy metal concentrations in the wastewater by optimizing the detection conditions (Figure 1). Therefore, stripping voltammetry using a three-electrode system was selected for heavy metal detection in pig farm wastewater (Figure S1). 

**Figure 2:**
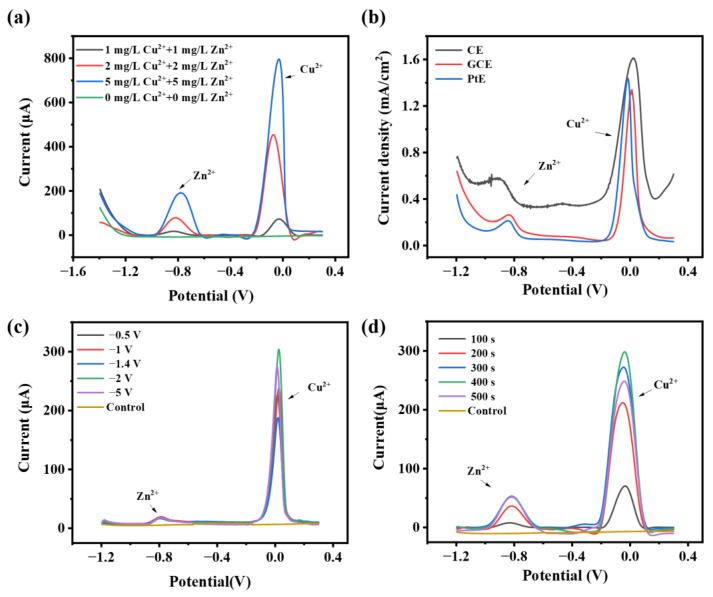
(**a**) Detection of different concentrations of Cu^2+^ and Zn^2+^ with DPSV using GCE. (**b**) Detection of 1 mg/L Cu^2+^ and 1 mg/L Zn^2+^ with different electrodes. (**c**) Detection of 1 mg/L Cu^2+^ and 1 mg/L Zn^2+^ under different enrichment voltages with GCE. (**d**) Detection of 1 mg/L Cu^2+^ and 1 mg/L Zn^2+^ under different enrichment times with GCE.

The working electrode was the essential sensing element for electrochemical sensors, so it was necessary to choose appropriate working electrodes first. Thus, three common working electrodes were chosen for comparison: the GCE electrode, the carbon rod electrode (CE), and the platinum wire electrode (PtE). The current density, peak spacing, peak shape, and other factors of different electrodes for different concentrations of heavy metals were compared. Compared with other electrodes, the detection of GCE could appear as characteristic peaks with obvious peak shapes and a linear relationship within a certain concentration range (Figure 2a and Figure S3). The surface morphology of the GCE after enrichment with heavy metal ions was observed by SEM (Figure S2), which showed nanoparticles anchored on the electrode surface. The EDS analysis indicated that the electrode surface was successfully enriched with a large number of metal particles (Figure S2), which confirmed the feasibility of the stripping voltammetry method. DPSV, SWSV, and LSV were performed for electrochemical detection of heavy metals. The responses of the different methods applied to the simultaneous detection of various concentrations of Cu^2+^ and Zn^2+^ were determined (Figure 2b and Figure S4). When detecting the same concentration of Cu^2+^ and Zn^2+^, DPSV showed the highest characteristic peak current and the best peak shape, with a good linear relationship among the three methods [40,41]. The current peak positions of Cu^2+^ and Zn^2+^ were located around 0 V and −0.8 V, respectively. Whereas the linear change of the current of Cu^2+^ at 2 mg/L and 5 mg/L using the SWSV method was not obvious, in addition to the problem of Zn^2+^ peak shift, GCE and DPSV were chosen for the following experiments. Therefore, in the following experiment, GCE and DPSV were selected. In addition, pulse width and pulse period were the main influential parameters in DPV detection. We optimized the conditions using a solution containing the same Cu^2+^, and Figure S5 shows the detection results for different DPV parameters. Comparing the results from the shape, width, and size of the current peaks, a pulse period of 0.03 s and a pulse width of 0.6 s were chosen as the detection conditions for this experiment.

The effect of deposition potential in the range of −0.5~−5 V potential on the enrichment of heavy metals was investigated (Figure 2c). In addition, when the potential was more negative than −1 V, hydrogen production occurred and bubbles were generated on the electrode surface during the enrichment process. The results showed that the current signals of Zn^2+^ did not change obviously with the change of voltage, but the current signals of Cu^2+^ produced a significant change, and the peak current of Cu^2+^ increased in the range of −0.5~−2 V, reached the maximum value at −2 V, and then decreased slowly. The current signal of Cu ions had a maximum of about 300 μA. Therefore, the deposition potential of −2 V was chosen as the optimum condition, and further measurements were carried out at the deposition potential of −2 V.

The enrichment time could significantly affect the measurement results. To investigate the effect of enrichment time, the effect of 100~500 s was investigated in a solution containing 1 mg/L Cu^2+^ and Zn^2+^ (Figure 2d). The results showed that the peak current of metal ions increased in the first 400 s with an increase in deposition time, and the current decreased after 500 s. The current responses of Cu^2+^ and Zn^2+^ were around 295 μA and 55 μA, respectively. Too many metal ions adsorbed on the electrode surface for too long an enrichment time might result in the shedding of the enriched metal as well as hindering the dissolution of the inner metal, so 400 s was chosen as the enrichment time. 

### 3.2. Effect of Cd^2+^ on the Electrochemical Detection of Cu^2+^ and Zn^2+^

**Figure 3:**
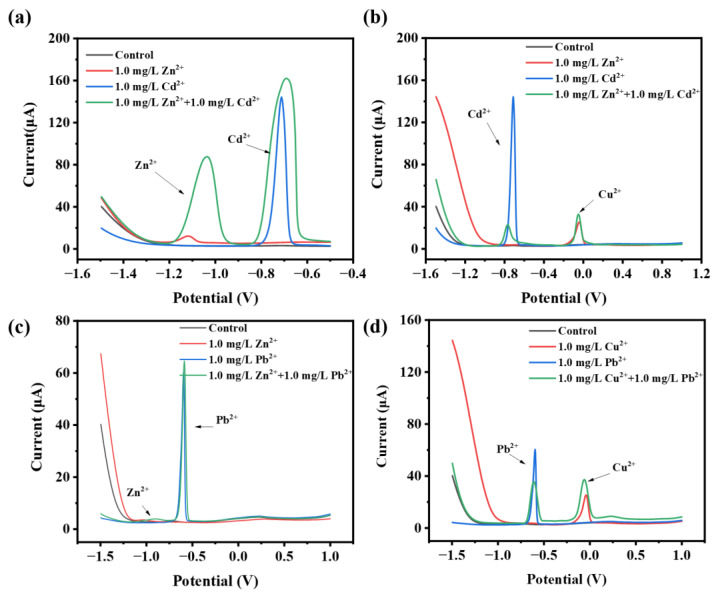
The results of DPSV with the addition of different heavy metal ions. (**a**) 1 mg/L Zn^2+^ and 1 mg/L Cd^2+^; (**b**) 1 mg/L Cu^2+^ and 1 mg/L Cd^2+^; (**c**) 1 mg/L Zn^2+^ and 1 mg/L Pb^2+^; (**d**) 1 mg/L Cu^2+^ and 1 mg/L Pb^2+^.

Previous studies showed that cadmium modification had a beneficial impact on the electrochemical performance of specific cathode materials, such as LiCoO_2_ [42], LiFePO_4_ [43], Li_2_FeSiO_4_ [44], and LiNi_0.8_Co_0.15_Al_0.05_O_2_ [45]. Attempts were made to use cadmium as an electrochemically active element in electrode materials for joint modification to improve electrochemical performance. Therefore, in this study, additional Cd^2+^ was added to samples to explore their effect on the electrochemical response (Figure 3a,b). The results showed that adding a certain concentration of Cd^2+^ could significantly improve the current signal of Zn^2+^. The current value of Zn^2+^ increased linearly when adding 0.5~1 mg/L Cd^2+^, and the current signal of Zn^2+^ reached a maximum value of 84 mA when adding 1 mg/L Cd^2+^, which increased the signal by eight times. However, the current value of Zn^2+^ did not continue to increase when adding 2 mg/L Cd^2+^, indicating that the concentration of Cd^2+^ might be saturated at the concentration of 1 mg/L. Excessive concentration would inhibit the enrichment and current response of Zn. Adding Pb^2+^ did not enhance the current response (Figure 3c,d and Figure S6), which further demonstrated that the enrichment of Cd^2+^ might have a catalytic effect that enhances the output current signal of Zn^2+^. The explanation for the enhanced detection may be related to interactions between different elements, such as the formation of new compounds, which can cause additional waves, signal depression or enhancement, etc. [46]. The results indicated that Cd^2+^ addition would be useful for improving the detection of Zn^2+^ and also highlighted that Cd^2+^ might be an interference ion for Zn^2+^ once they coexisted.

### 3.3. Selection and Optimization of the Digestion Method

**Figure 4:**
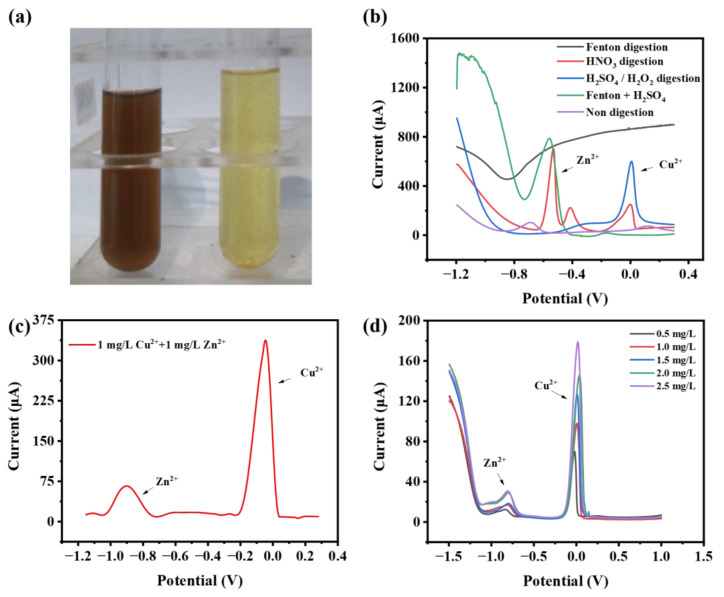
(**a**) The photograph of pig farm wastewater before and after digestion. (**b**) The DPSV results of pig farming wastewater (with high Cu^2+^ and Zn^2+^) after different digestion methods. (**c**) Characteristic peak currents of Cu^2+^ and Zn^2+^ after digestion with nitric acid (pig farming wastewater with no detectable heavy metal ions but with exogenously added 1 mg/L Cu^2+^ and 1 mg/L Zn^2+^). (**d**) The results of DPSV of pig farm wastewater (with no detectable heavy metal ions) spiked with different concentrations of Cu^2+^ and Zn^2+^.

In the actual sample testing, aquaculture wastewater’s high turbidity and high organic content were not suitable for direct electrochemical detection. Samples needed to be digested to convert the metal elements’ various states and valence states to be detected into the same valence state, remove organic matter, and reduce turbidity to eliminate their interference. The main methods of digestion were wet digestion, microwave digestion, dry digestion, and electrochemical oxidation digestion. Wet digestion by using strong oxidizing acids or oxides to oxidize the substance was used here. Meanwhile, the main digestion solutions selected were concentrated nitric acid, concentrated sulfuric acid, or hydrogen peroxide. In addition, Fenton’s advanced oxidation method was also used for comparison. Figure 4a shows the representative color changes of the pig farm wastewater before and after the digestion. After filtration, the digestion process resulted in a clear solution without precipitation.

Figure 4b shows the detection results of the actual samples using different digestion methods. As shown in the results, no characteristic peaks of the metal ions appeared after measuring in the farm wastewater digested by Fenton’s reagent, while characteristic peaks of Cu^2+^ and Zn^2+^ were detected at around 0 V and −0.6 V in the solution digested by Fenton’s reagent with the addition of sulfuric acid, which indicated that the Fenton reagent itself could not efficiently release the heavy metals, which mainly relied on the action of the strong acid. Compared with sulfuric acid, the characteristic peaks and the current response of Cu^2+^ and Zn^2+^ after nitric acid digestion were more obvious, and the different peak results might be due to the effect of other ions in the sample. Finally, nitric acid was chosen as the digestion solution for the pre-treatment of the pig farm wastewater (Figure 4c). Calibration curves were obtained based on the signal response of the sensing system to 0.5~2.5 mg of Cu^2+^ and Zn^2+^ in wastewater (Figure 4d and Figure S7). With good linear results (r = 0.99, 0.91), the LODs of Cu^2+^ and Zn^2+^ were 9.3 μg/L and 45.3 μg/L, respectively (Figure S7). Then, the spiking recoveries of the electrochemical method were tested. As shown in Table 1, the spiked recoveries obtained ranged from 92% to 99% at spiked concentrations of 1.5~10.5 mg/L.

### 3.4. Simutaneous Detection of Cu^2+^ and Zn^2+^ in Real Pig Farm Wastewater

Meanwhile, the concentrations of Cu^2+^ and Zn^2+^ in pig farm wastewater samples were also examined. The actual pig farm wastewater samples were taken from a pig farm in Huaian, Jiangsu Province. ICP-MS was applied to check the possible coexistence of Cd^2+^ and Zn^2+^ in this wastewater before electrochemical sensing (a corrected calibration curve should be used if Cd^2+^ is present). It was shown that no significant Cd^2+^ was detected in these samples. Then, the developed electrochemical method was used to monitor the heavy metal ions of Cu^2+^ and Zn^2+^ in these real samples. The results of the concentration in the wastewater detected by the electrochemical method were also compared with the results from ICP-MS. When comparing the results of ICP-MS with the method of the present study on the actual water samples, the range of the relative deviation values was 8.6~14.7% (Table 2). These results suggested that the electrochemical method developed here had the potential to quantify these heavy metal ions for practical applications.

## 4. Conclusions

In this study, simultaneous detection of Cu^2+^ and Zn^2+^ in pig farm wastewater was achieved by using an unmodified GCE electrode with anodic stripping voltammetry. The electrode, electrochemical method, and digestion method were all optimized, and the obtained LODs of Cu^2+^ and Zn^2+^ were 9.3 μg/L and 45.3 μg/L, respectively. The spiked recoveries of Cu^2+^ and Zn^2+^ in the samples ranged from 92% to 99%. In addition, it was found that adding Cd^2+^ to the solution could improve the electrochemical response of Zn^2+^, which increased the output signal by eight times. This work provided the possibility of electrochemical measurement for the detection of metal ions in pig farm wastewater and provided a new and simple tool for fast monitoring of the wastewater from pig farms.

## Figures and Tables

**Table 1:** Quantification results of distilled water spiked with authentic metal ions.

Sample	Cu^2+^ and Zn^2+^ Spiked (mg/L)	Detection of Cu^2+^ and Zn^2+^ after Spiking (mg/L)	Recovery Rate
1	1.5/1.5	1.38/1.40	92%/93%
2	2.5/2.5	2.45/2.41	98%/96%
3	5.5/5.5	5.42/5.36	99%/97%
4	10.5/10.5	10.42/10.38	99%/99%

**Table 2:** Quantification results of real pig farm wastewater.

Sample	Concentration Detected by ICP (mg/L)	Concentration Detected by Electrochemical (mg/L)	Deviation
1	6.54/7.34	7.32/8.23	11.9%/12.2%
2	4.34/5.68	4.98/6.45	14.7%/13.6%
3	5.53/6.32	4.73/5.54	14.4%/12.3%
4	7.36/9.42	8.20/10.23	11.4%/8.6%

## Data Availability

The data presented in this study are available on request from the corresponding author.

## Supplementary Materials

The following supporting information can be downloaded at: https://www.mdpi.com/article/10.3390/s24082475/s1, Figure S1: Schematic diagram of the three-electrode system; Figure S2: SEM images and EDS analysis of the GCE electrodes; Figure S3: The results of electrochemical sensing under different conditions; Figure S4: The results of different electrochemical methods with the addition of different heavy metal ions. Figure S5: The effects of different DPV parameters; Figure S6: The results of DPSV with the addition of different heavy metal ions; Figure S7: Calibration curve for DPSV detection of Cu^2+^ and Zn^2+^.
