# Supplementary material for: Simultaneous Electrochemical Detection of Cu2+ and Zn2+ in Pig Farm Wastewater
Source: Sensors (Basel). 2024 Apr 12;24(8):2475. doi: 10.3390/s24082475 (PMC11053575; doi:10.3390/s24082475)
Supplement: Supplementary file 1 [file sensors-24-02475-s001.zip › sensors-2948654-supplementary.pdf]

Supplementary Information

Simultaneous Electrochemical Detection of Cu^{2+} and Zn^{2+} in Pig Farm Wastewater

Jia-Xin Du[#], Yang-Hao Ma[#], Said Nawab, Yang-Chun Yong*

Biofuels Institute and Institute for Energy Research, School of Environment and Safety Engineering,

Jiangsu University, 301 Xuefu Road, Zhenjiang 212013, China

#Equal contribution

**Corresponding author, Email:ycyong@ujs.edu.cn*

1. Electrode reaction for anodic stripping voltammetry

The electrode reactions involved in this study are shown below:

$$Zn^{2+}_{(aq)} + 2e^{-} \leftrightarrow Zn_{(s)}$$

$$Cu^{2+}_{(aq)} + 2e^{-} \leftrightarrow Cu_{(s)}$$

The metal ion is dissolved in the solution; during the accumulation step, it is deposited on the surface of a solid planar electrode in the form of a film on the GCE. During the stripping step, a potential sweep in an anodic direction is applied, and an electrochemical response is recorded because of the dissolution of the film.

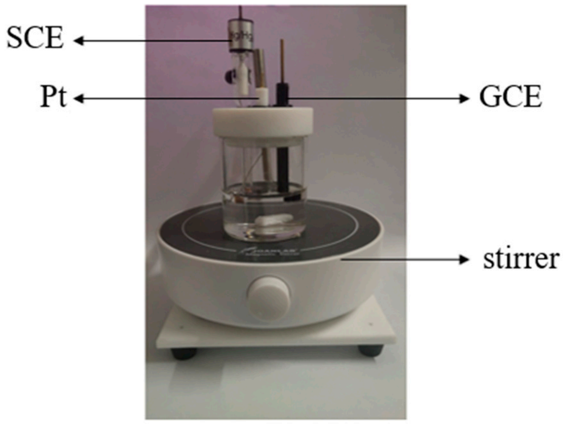

Figure S1. Schematic diagram of the three-electrode system. Working electrode, GCE; reference electrode, saturated calomel electrode; counter electrode, platinum wire electrode.

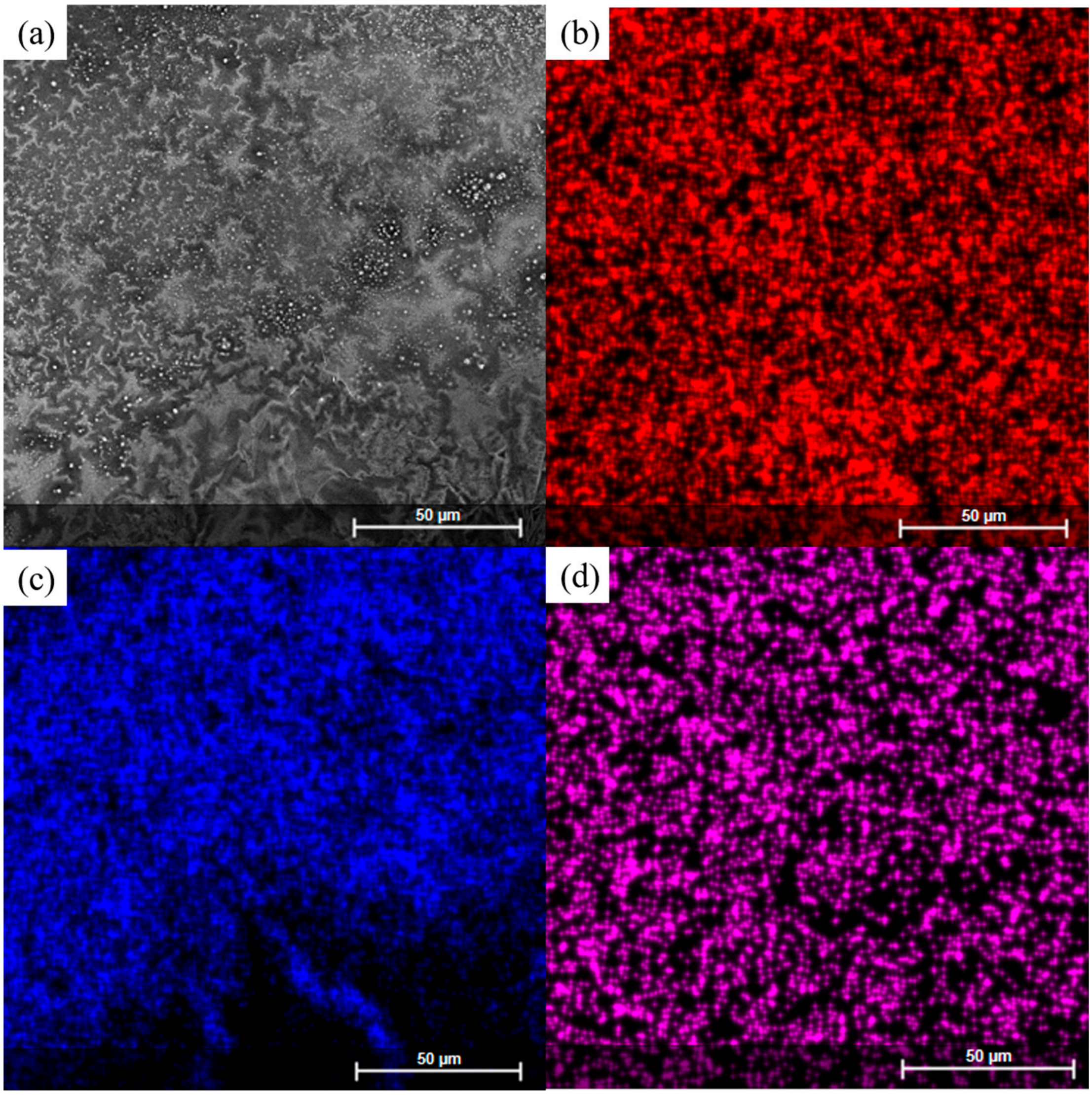

Figure S2. SEM image of (a) the GCE electrode after metal ions deposition and the energy dispersive spectrometer (EDS) analysis of (b) carbon, (c) copper, (d) znic in the selected area.

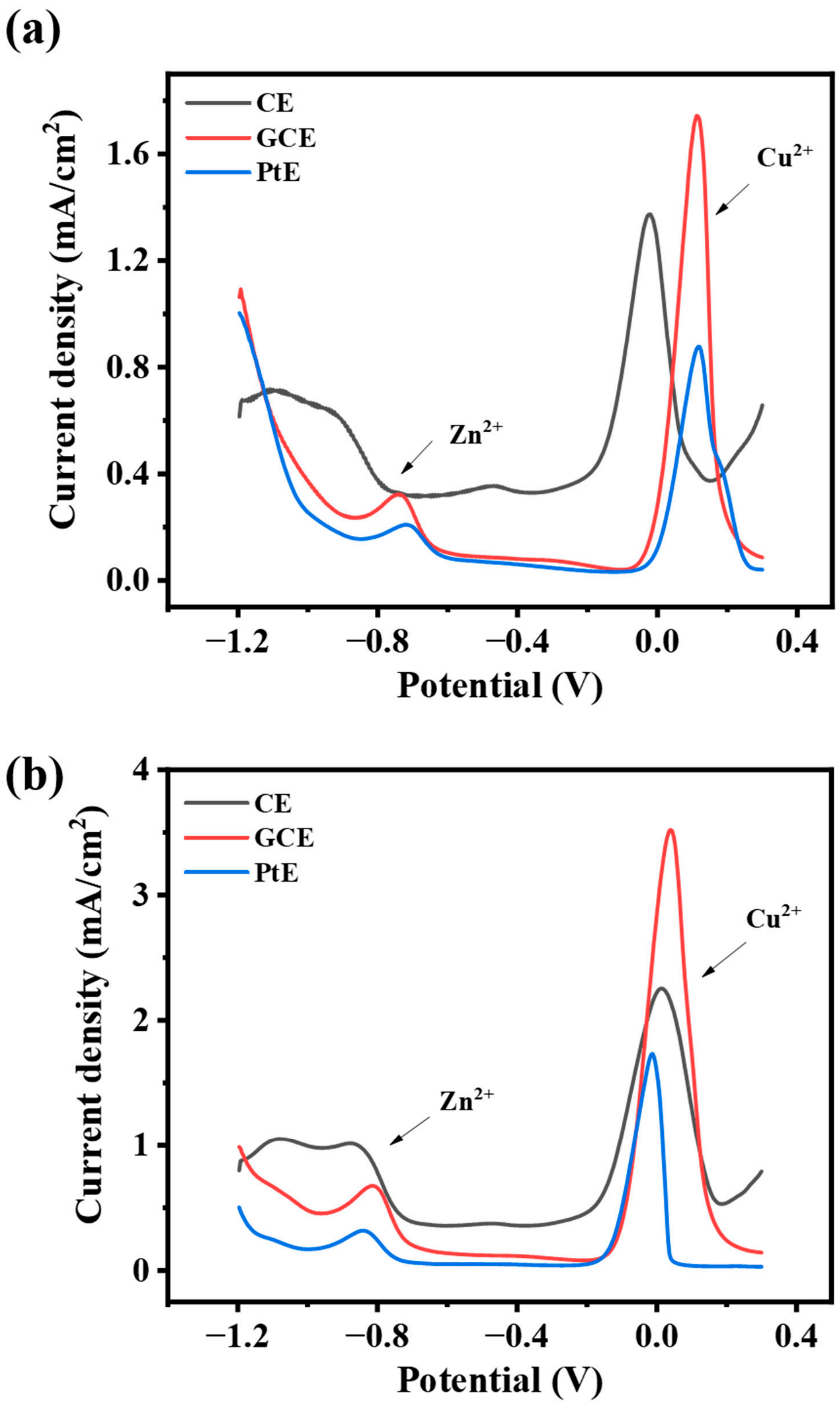

Figure S3. The results of electrochemical sensing under different conditions. (a) The results of 2 mg/L Cu^{2+} and 2 mg/L Zn^{2+} detected at different electrode conditions. (b) The results of 5 mg/L Cu^{2+} and 5 mg/L Zn^{2+} detected at different electrode conditions.

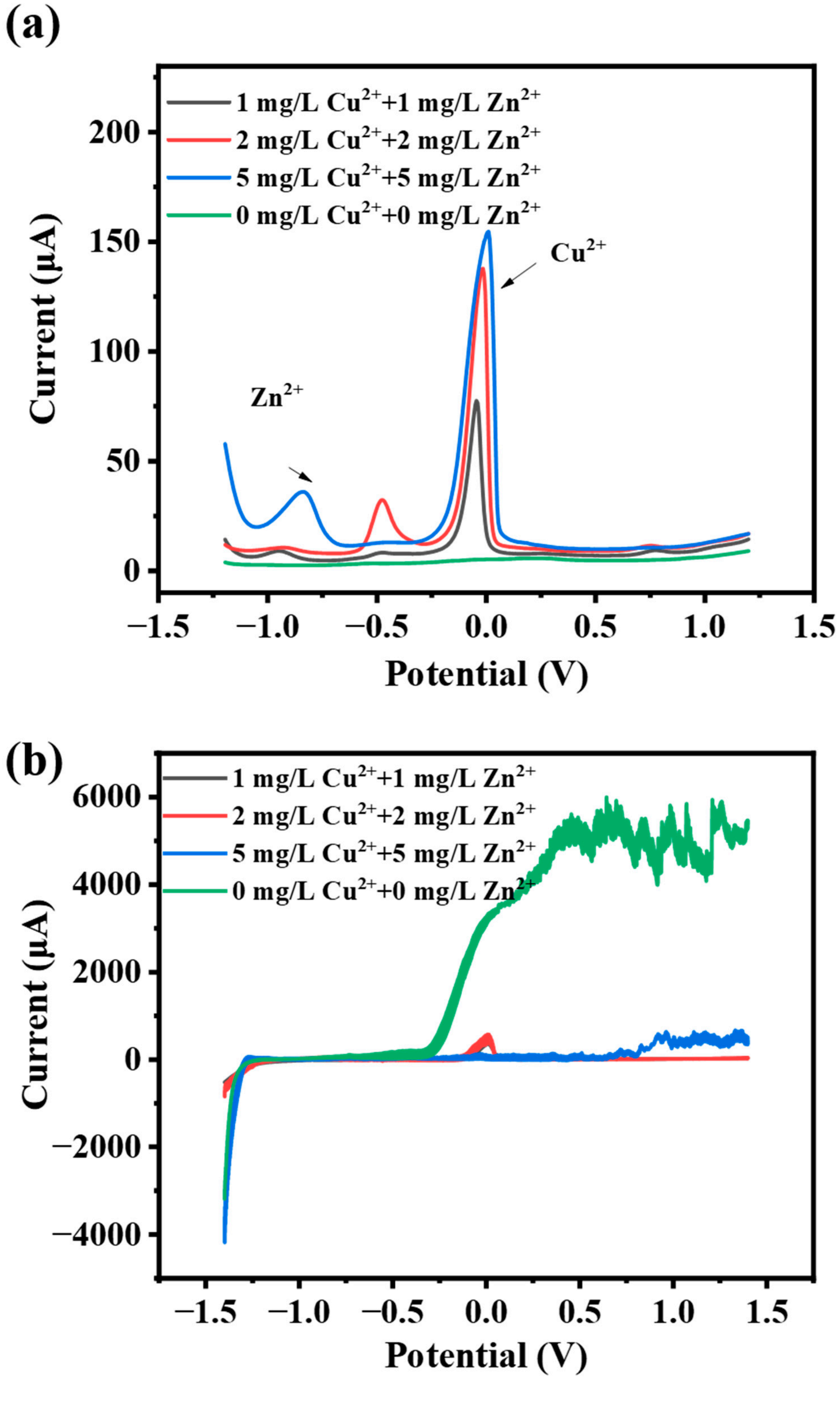

Figure S4. The results of different electrochemical methods with the addition of different heavy metal ions. (a) SWSV, (b) LSV.

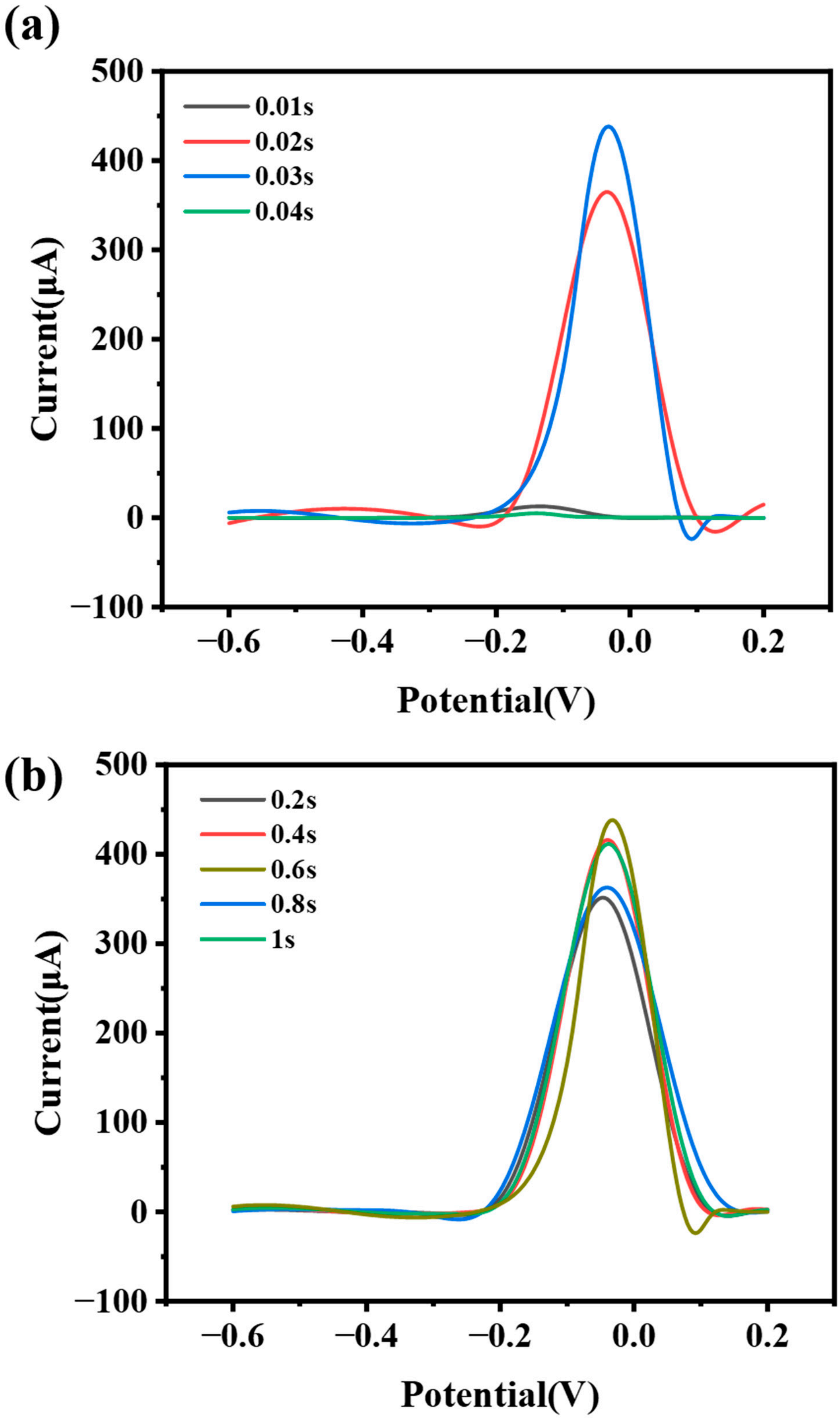

Figure S5. The effects of different DPV parameters. (a) Pulse period, (b) pulse width.

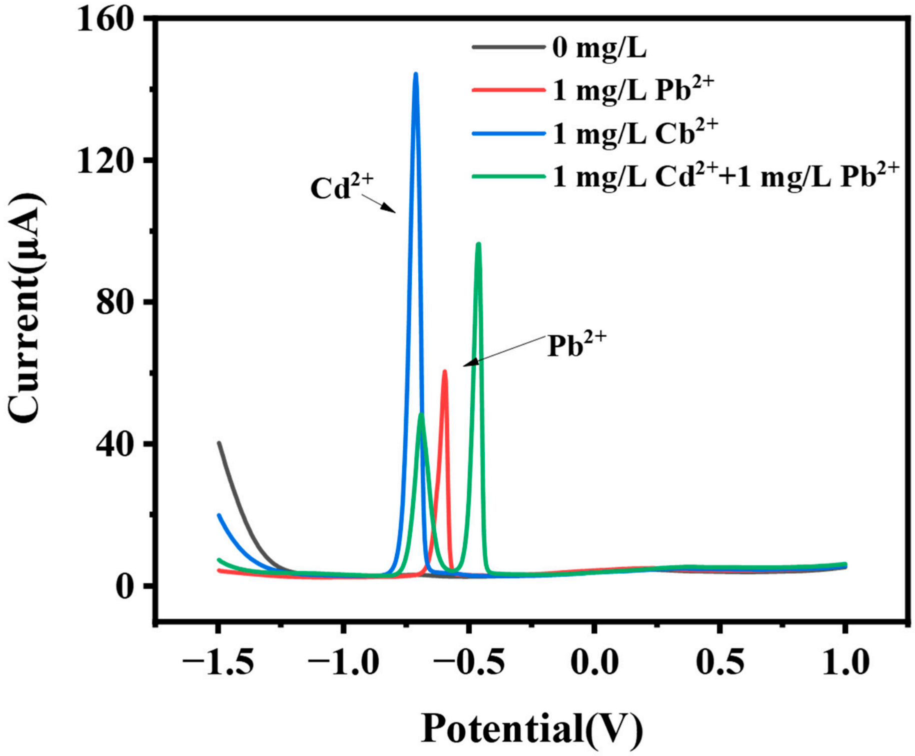

Figure S6. The results of DPSV with the addition of different heavy metal ions (Pb^{2+} and Cd^{2+}).

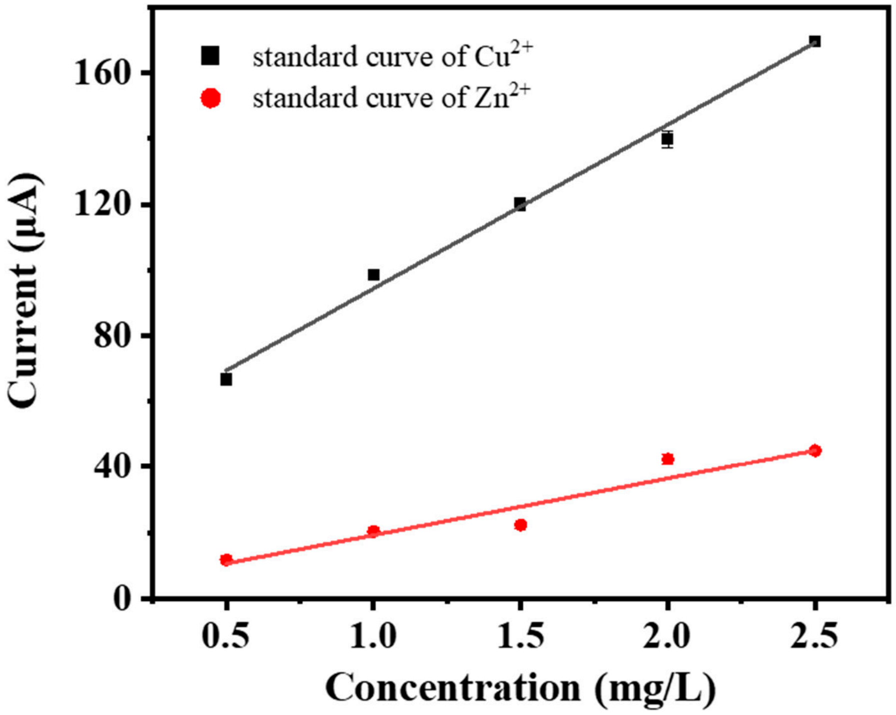

Figure S7. Calibration curve for DPSV detection of Cu^{2+} and Zn^{2+}.
